# Supplementary figures and images for: Long-Chain Acyl-CoA Synthetases Promote Poplar Resistance to Abiotic Stress by Regulating Long-Chain Fatty Acid Biosynthesis
Source: Int J Mol Sci. 2022 Jul 29;23(15):8401. doi: 10.3390/ijms23158401 (PMC9369374; doi:10.3390/ijms23158401)

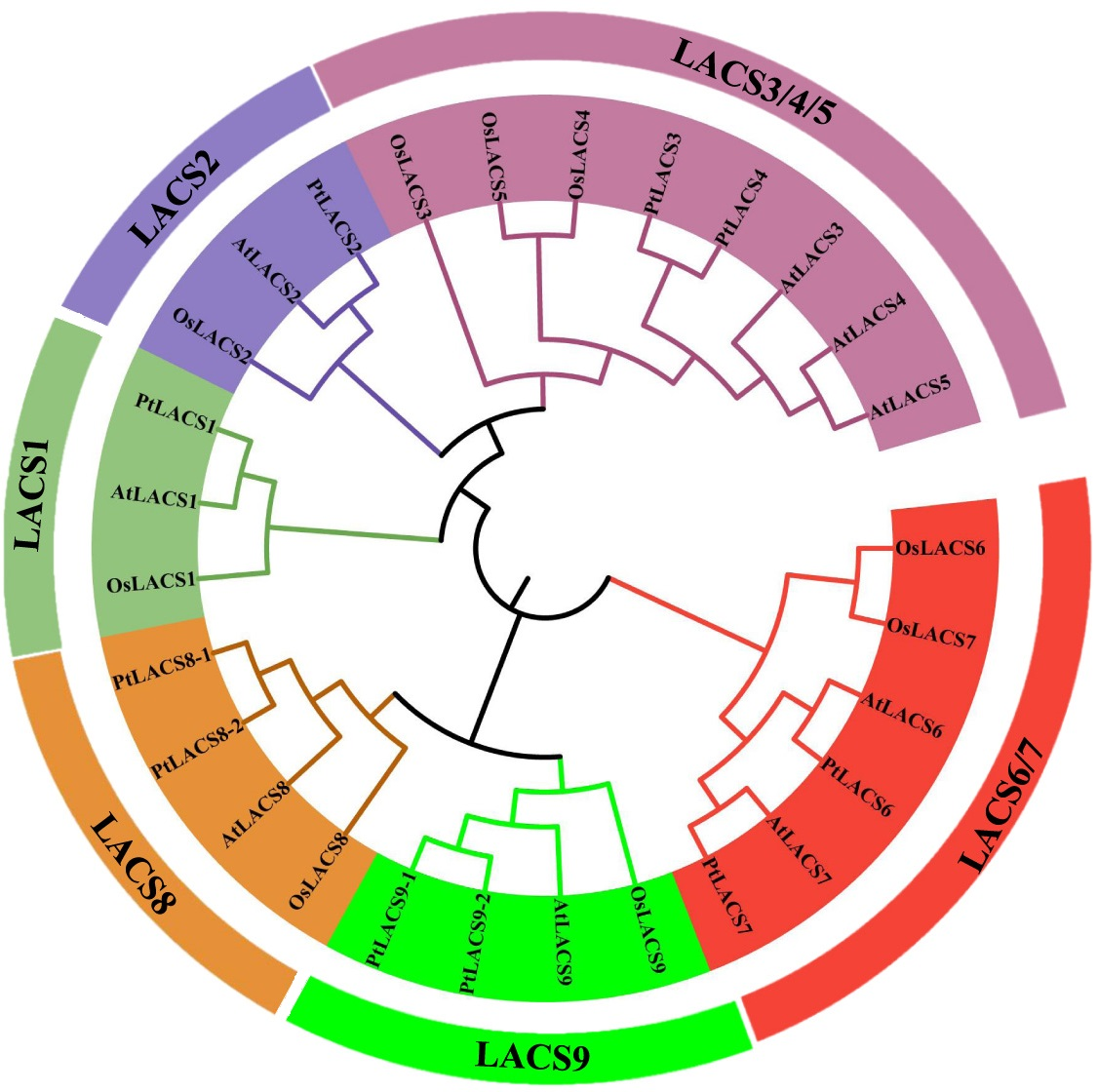

Supplement: Supplementary file 1 [file ijms-23-08401-s001.zip › Supplementary Figure S1.tif]

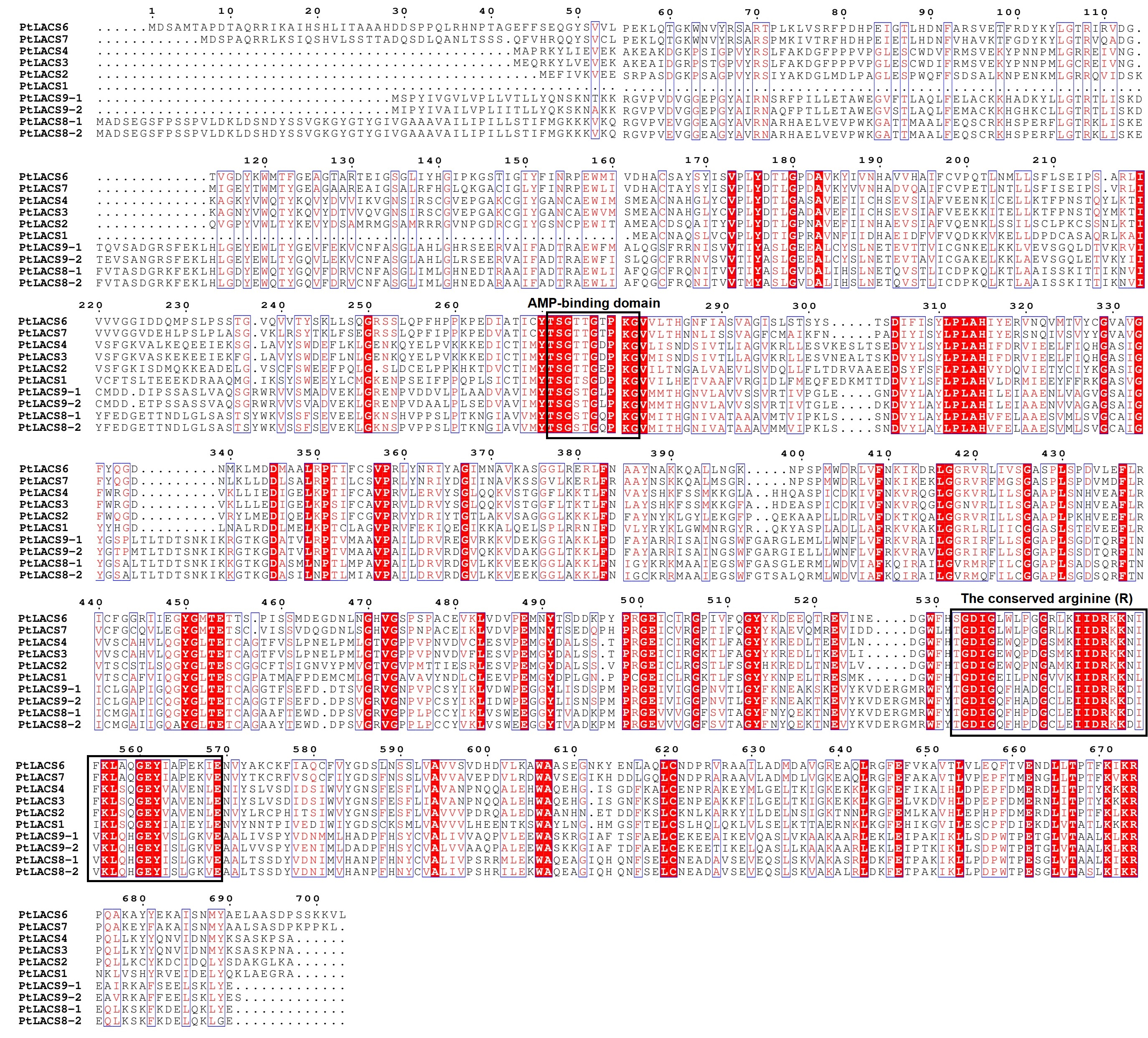

Supplement: Supplementary file 1 [file ijms-23-08401-s001.zip › Supplementary Figure S2.tif]

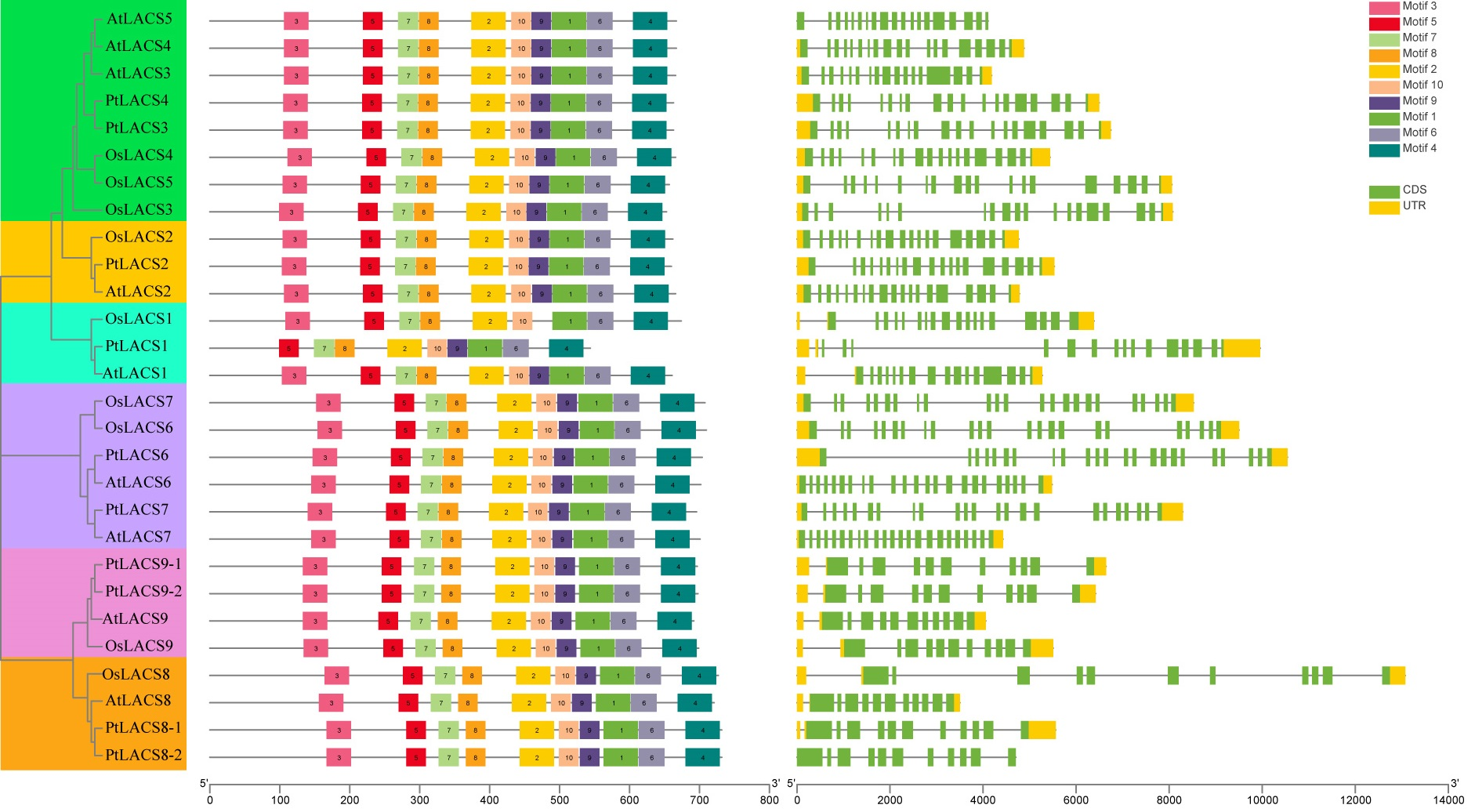

Supplement: Supplementary file 1 [file ijms-23-08401-s001.zip › Supplementary Figure S3.tif]

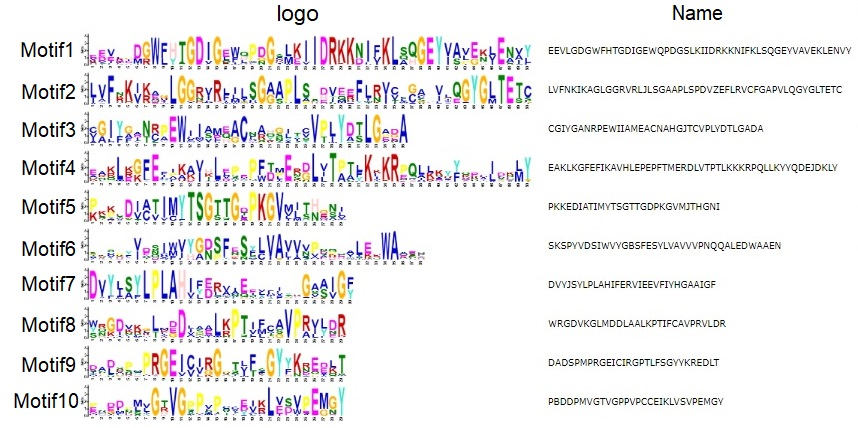

Supplement: Supplementary file 1 [file ijms-23-08401-s001.zip › Supplementary Figure S4.tif]

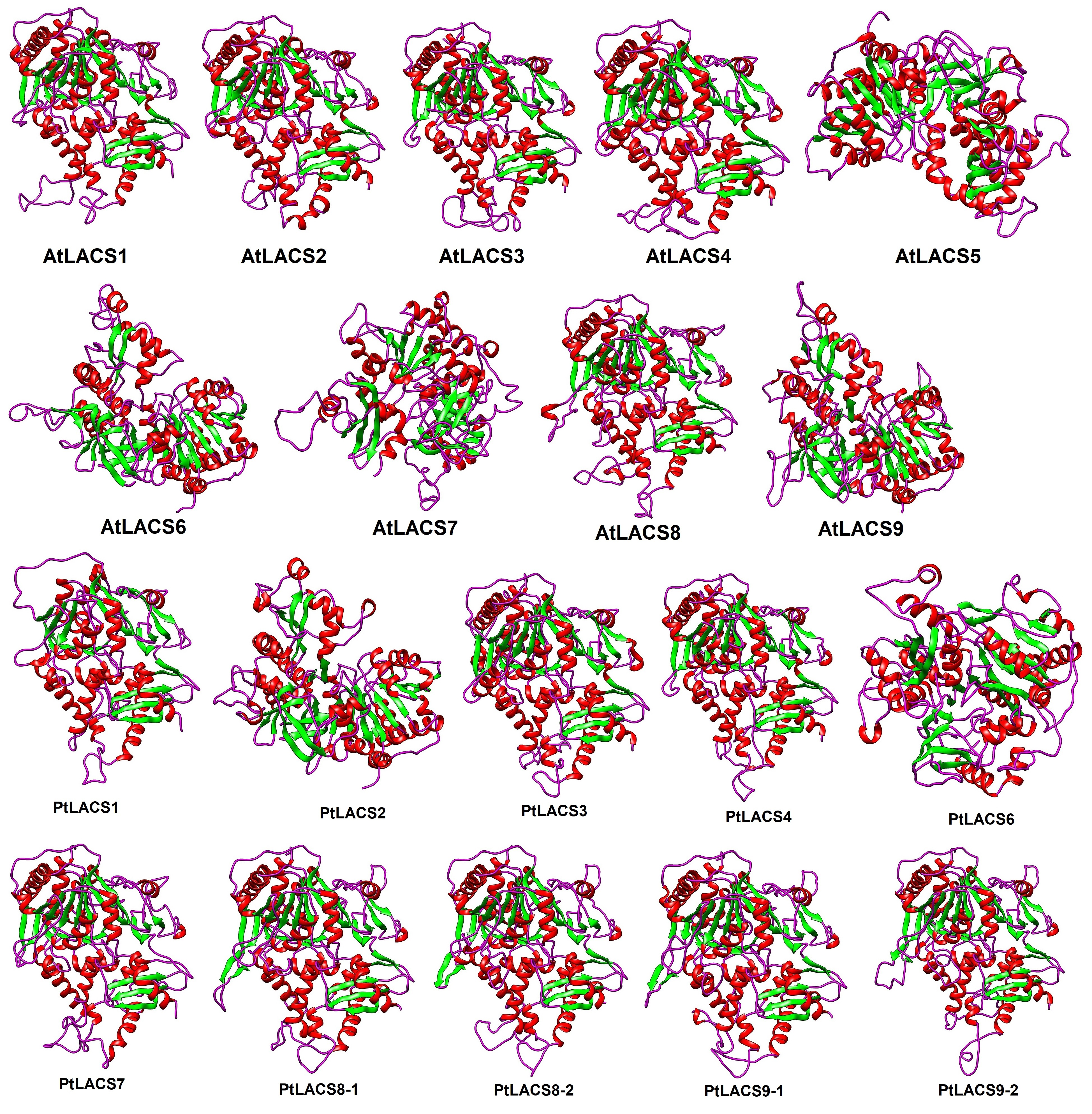

Supplement: Supplementary file 1 [file ijms-23-08401-s001.zip › Supplementary Figure S5.tif]

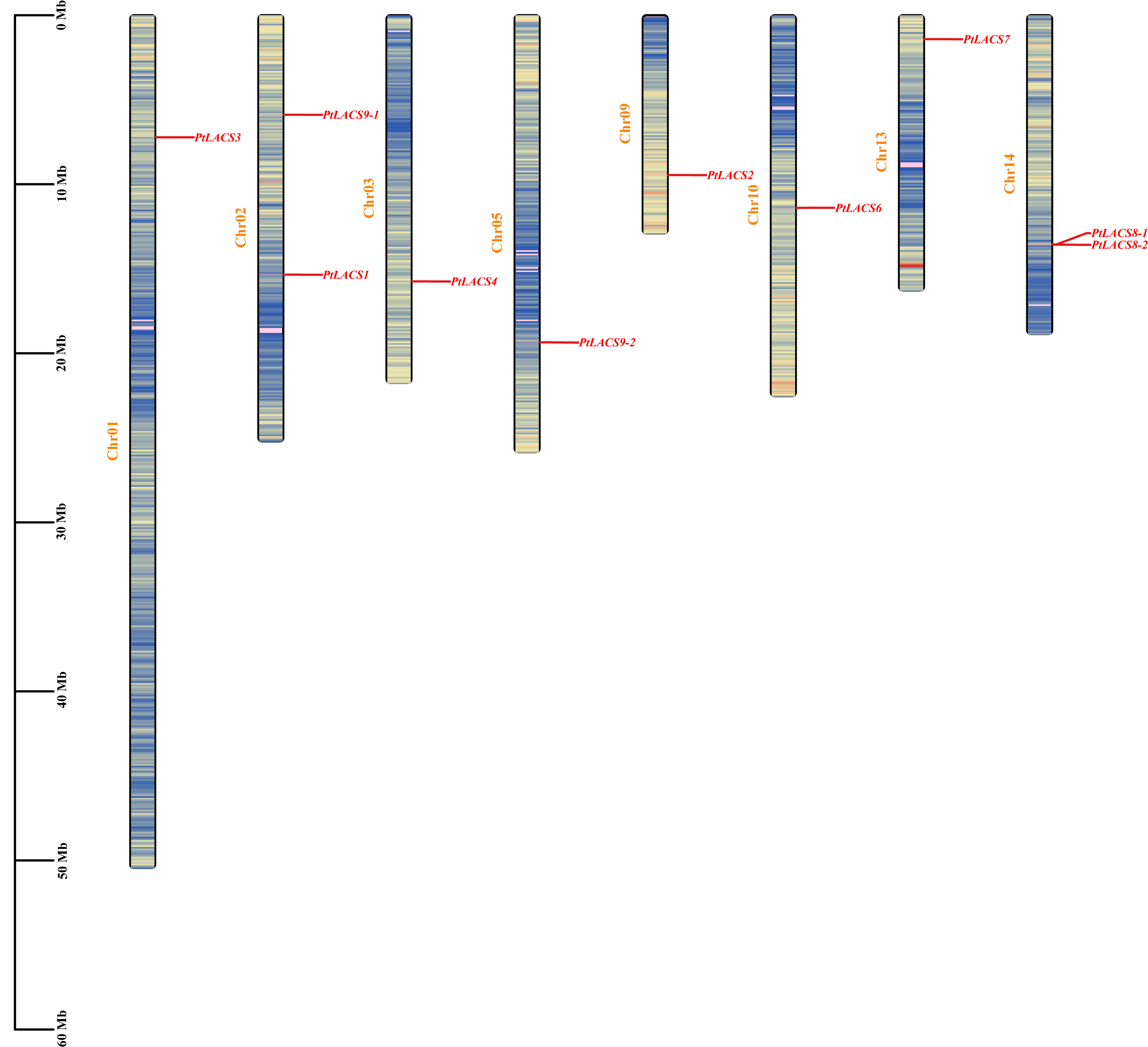

Supplement: Supplementary file 1 [file ijms-23-08401-s001.zip › Supplementary Figure S6.tif]

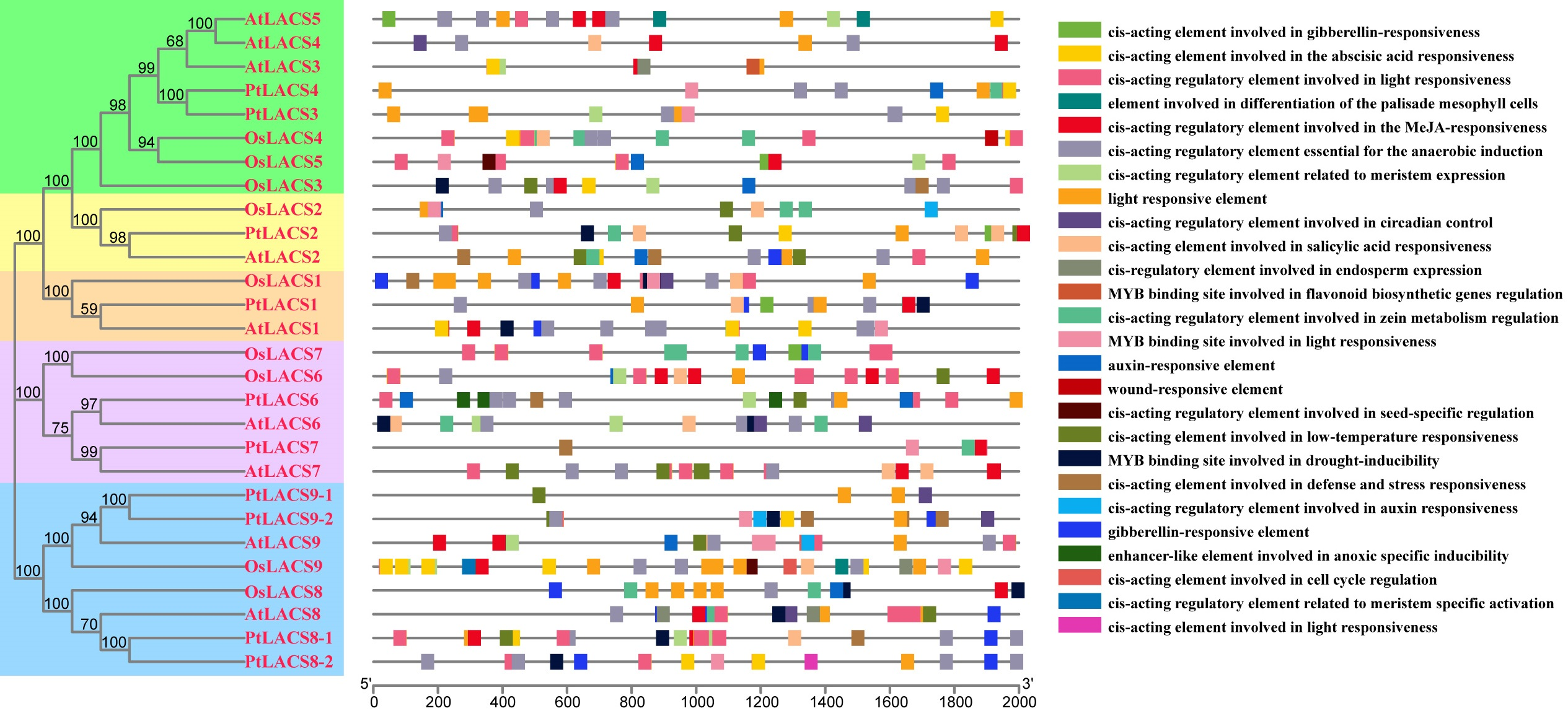

Supplement: Supplementary file 1 [file ijms-23-08401-s001.zip › Supplementary Figure S7.tif]

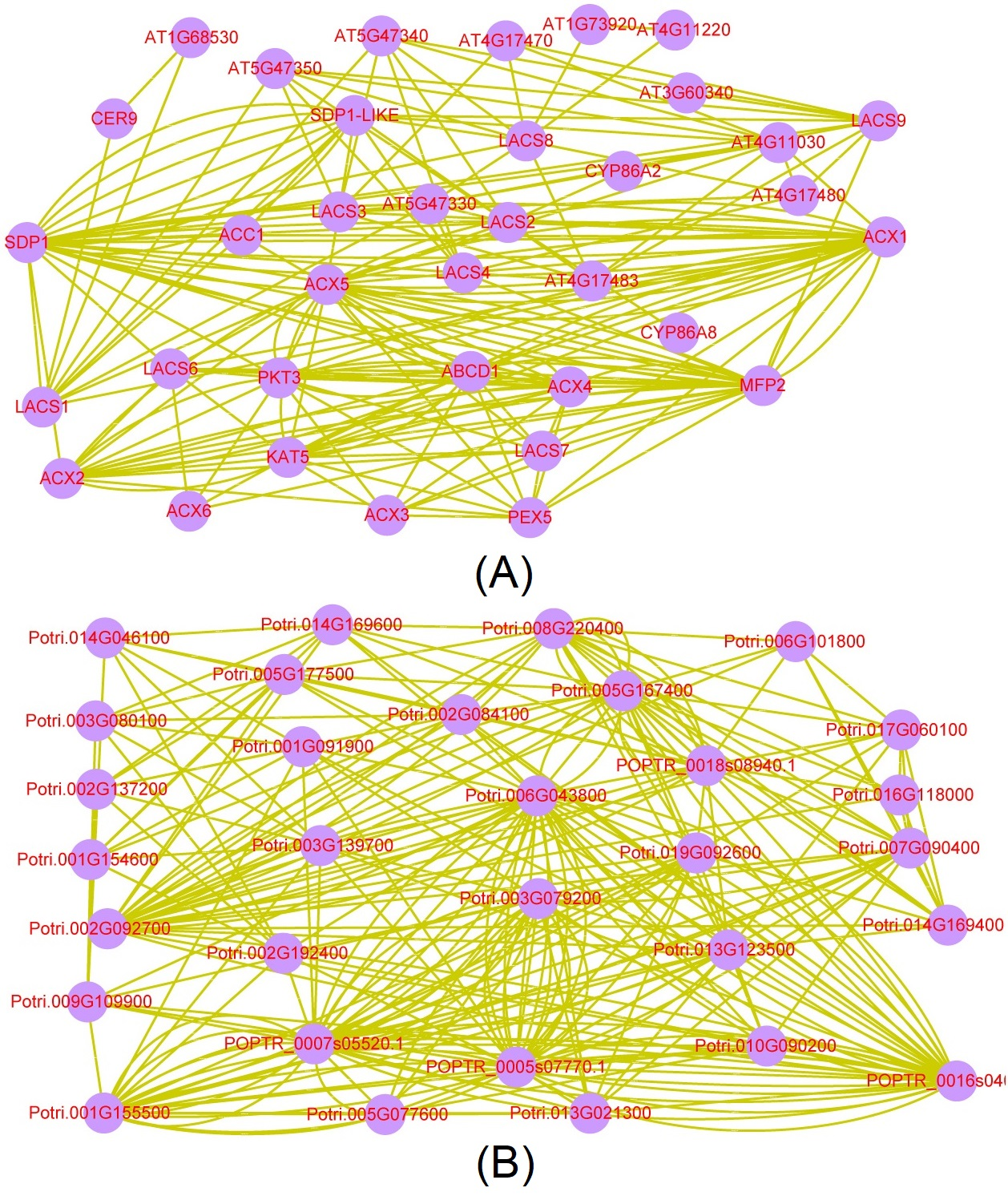

Supplement: Supplementary file 1 [file ijms-23-08401-s001.zip › Supplementary Figure S8.tif]
